# Application of Quantitative PCR (qPCR) for the Detection of Psittacine Beak and Feather Disease Virus (PBFDV) in Air Conditioning Systems: A Model Study from a Veterinary Hospital in Thailand

**DOI:** 10.3390/vetsci13050498

**Published:** 2026-05-20

**Authors:** Benchapol Lorsunyaluck, Juthanan Charachit, Sasipat Putsetkun, Natapol Pumipuntu

**Affiliations:** 1 Panalai Veterinary Hospital, Pakkret, Nonthaburi 11120, Thailand; 2 Akkhraratchakumari Veterinary College, Walailak University, Nakhon Si Thammarat 80161, Thailand; 3 Faculty of Science, Naresuan University, Phitsanulok 65000, Thailand; 4 One Health Research Unit, Mahasarakham University, Maha Sarakham 44000, Thailand; 5 Faculty of Veterinary Sciences, Mahasarakham University, Maha Sarakham 44000, Thailand

**Keywords:** psittacine beak and feather disease virus, circovirus, quantitative PCR, environmental contamination, air conditioning systems, veterinary hospital

## Abstract

Psittacine beak and feather disease virus (PBFDV), a member of the family *Circoviridae*, is a major pathogen affecting psittacine birds worldwide; however, its potential for airborne dissemination in veterinary environments remains poorly understood. This study aimed to investigate PBFDV contamination in air conditioning systems within a veterinary hospital and to compare the distribution and levels of viral load across different functional areas. Environmental swab samples were collected from 17 air conditioning units located in examination rooms, surgical suites, wards, and laboratory areas. Viral nucleic acids were extracted and analyzed using quantitative polymerase chain reaction (qPCR) on the Genesig q16 (Version 4) platform. PBFDV DNA was detected in multiple units, with viral loads ranging from <10 to >25,000 PBFDV genome copies per qPCR reaction. The highest levels were observed in an examination room (26,172 copies) and a surgical room (25,730 copies), whereas several locations showed low or negligible contamination (<100 copies). These findings indicate that air conditioning systems may act as a possible environmental contamination pathway and potential sources of viral dissemination within clinical settings. The results underscore the importance of routine environmental monitoring and targeted disinfection strategies. As a preliminary model, this study provides baseline data to support the development of effective biosecurity measures to reduce the risk of airborne transmission of PBFDV in veterinary facilities.

## Simple Summary

Psittacine beak and feather disease (PBFD) is a serious viral disease affecting parrots and other psittacine birds, often leading to feather loss, beak abnormalities, and weakened immunity. The virus can spread not only through direct contact between birds but also through contaminated environments, including airborne particles such as feather dust. In this study, we investigated the presence of the virus in air conditioning systems within a veterinary hospital using quantitative PCR (qPCR). The results showed that viral DNA was detected in all the sampled air conditioning units, with higher levels found in areas where exotic birds were treated. This suggests that air circulation systems may play a role in distributing viral particles within the hospital environment. These findings highlight the importance of environmental monitoring and proper cleaning and air management practices to reduce the risk of infection. This approach may also be useful for controlling other airborne pathogens in veterinary and clinical settings.

## 1. Introduction

Psittacine beak and feather disease (PBFD) is regarded as one of the most important infectious diseases affecting psittacine birds worldwide [1]. The disease is caused by Psittacine beak and feather disease virus (PBFDV), a small non-enveloped single-stranded DNA virus belonging to the family *Circoviridae* [2]. Since its first description in cockatoos during the 1970s, PBFDV has been reported in numerous psittacine species, including macaws, African grey parrots, Eclectus parrots, ringneck parakeets, and lovebirds [3]. The infection is most commonly recognized in juvenile birds and is associated with progressive feather abnormalities, beak deformities, and immunosuppression, which may increase susceptibility to secondary infections [4]. Transmission of PBFDV can occur through direct contact with infected birds as well as indirect exposure to contaminated feather dust, aerosols, feces, and fomites [5]. Importantly, the virus demonstrates considerable environmental stability and may persist in contaminated facilities for prolonged periods, thereby contributing to indirect dissemination and ongoing environmental contamination [1]. Because no specific antiviral therapy is currently available, disease control primarily relies on biosecurity measures, including routine molecular screening, environmental disinfection, and infection control practices [6,7,8]. Although PBFDV transmission among birds has been extensively documented, limited information is available regarding environmental contamination within veterinary hospital environments, particularly in relation to ventilation and air conditioning systems. Therefore, environmental surveillance may provide valuable insight into potential reservoirs and mechanisms contributing to viral dissemination in clinical settings [9].

Veterinary hospitals represent high-risk environments for PBFDV transmission, as diverse psittacine species are frequently brought together for diagnosis and treatment [3]. Air conditioning systems, which recirculate air throughout clinical facilities, may serve as a potential environmental source for viral particles if not properly maintained [10]. Detecting PBFDV contamination in these systems is therefore essential for evaluating biosecurity risks and developing effective infection control strategies [9]. In addition, quantitative polymerase chain reaction (qPCR) offers high sensitivity and specificity for the detection and quantification of viral genomes, making it a valuable tool for environmental monitoring [1]. Applying qPCR to assess PBFDV contamination in air conditioning systems provides critical baseline data for infection prevention in veterinary hospitals [11].

The objective of this study was to investigate the presence of PBFDV in air conditioning systems within an exotic veterinary hospital and to compare both the distribution and quantity of the virus across different rooms. In addition to detecting PBFDV, the study aimed to assess the level of environmental contamination, as indicated by viral load, among different functional areas, with particular emphasis on determining whether high-risk areas, such as clinical and surgical rooms, exhibit higher levels of contamination compared to non-clinical or support areas. This investigation was designed to provide baseline data on environmental contamination patterns in clinical settings, thereby supporting the development of appropriate infection control and biosecurity practices.

## 2. Materials and Methods

### 2.1. Sample Collection

**Figure 1:**
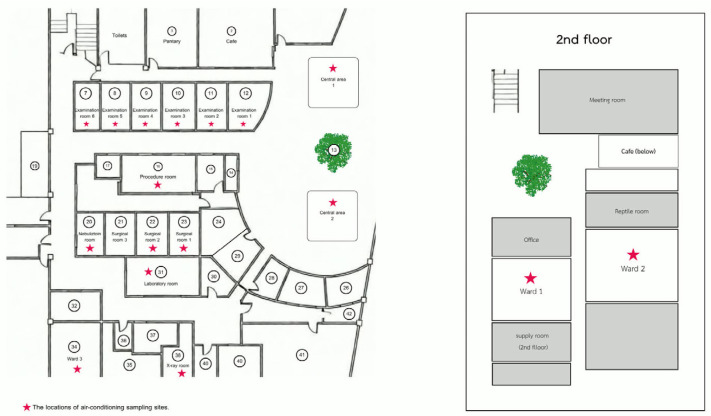
Floor plan of Panalai Veterinary Hospital showing the locations of air conditioning sampling sites.

This cross-sectional descriptive study was conducted at Panalai Veterinary Hospital, Nonthaburi, Thailand, which is a referral center with a high caseload of exotic pets, particularly avian species. Environmental samples were collected from air-conditioning systems within the hospital. A total of 17 indoor locations were included in the study. These locations included examination rooms, operating rooms, and wards. Diagnostic imaging rooms, treatment areas, the laboratory, and common spaces were also sampled. This study was designed as a preliminary cross-sectional environmental survey conducted within a single veterinary hospital. Therefore, all accessible air conditioning units within the facility (*n* = 17) were included in the study to provide baseline information regarding PBFDV environmental contamination. The spatial distribution of all sampled rooms is shown in Figure 1. Sampling focused specifically on condensate trays of air-conditioning units, which are known to accumulate airborne particulate matter and microorganisms.

Sterile cotton swabs were used to sample the surface of each condensate tray. Each swab was placed into a sterile 1.5 mL microcentrifuge tube containing 250 µL of sterile water, after which the swab was discarded. Samples were sealed, labeled, and stored at −20 °C until nucleic acid extraction. The veterinary hospital routinely performed cleaning and maintenance of air conditioning systems every Monday as part of standard environmental hygiene practices. Therefore, residual PBFDV contamination may still persist within air conditioner components, particularly in condensate trays and internal surfaces, despite routine cleaning procedures. To maximize the likelihood of detecting accumulated environmental contamination under normal operational conditions, sample collection was intentionally conducted on the final day of the week (Sunday). At this time point, viral particles and organic debris were expected to accumulate following routine hospital activities throughout the week. This sampling strategy was designed to reflect residual environmental contamination within the air conditioning systems under routine operational conditions.

### 2.2. Viral Nucleic Acid Extraction

Viral nucleic acids were extracted from environmental swab samples using a column-based extraction method following the standard protocol described for the viral nucleic acid purification kit from FavorPrep™ Viral Nucleic Acid Extraction Kit (FAVORGEN, Pin Tung, Taiwan). Samples were centrifuged to collect the liquid at the bottom of the tube, and 140 µL of each sample was transferred into a new microcentrifuge tube. Subsequently, 560 µL of viral nucleic acid extraction (VNE) buffer was added, and the mixture was incubated at room temperature for 10 min. Absolute ethanol (560 µL) was then added and mixed thoroughly by vortexing.

The lysate was loaded onto a silica membrane spin column (VNE column) and centrifuged at 8000× *g* for 1 min. The flow-through was discarded, and the loading step was repeated until the entire sample had passed through the column. The column was then washed with 500 µL of wash buffer 1 and 750 µL of wash buffer 2, with centrifugation at 8000× *g* for 1 min after each wash step. A final high-speed centrifugation at 18,000× *g* for 3 min was performed to remove residual wash buffer.

Viral nucleic acids were eluted by adding 50 µL of RNase-free water preheated to 70 °C directly onto the membrane, followed by incubation for 1 min and centrifugation at 18,000× *g* for 1 min. The eluted nucleic acids were collected in a clean microcentrifuge tube and stored at −20 °C until further analysis by quantitative PCR (qPCR).

### 2.3. Quantitative Real-Time PCR (qPCR) and Viral Load Quantification

Quantitative real-time PCR was performed using the Genesig q16 Real-Time PCR instrument (PrimerDesign Ltd., Manchester, UK), a compact, integrated platform designed for rapid and sensitive detection of nucleic acids. The system utilizes hydrolysis probe-based detection chemistry and incorporates pre-optimized lyophilized reagents to ensure assay consistency and reproducibility. The assay targets a conserved region within the replication-associated protein (Rep) gene of the PBFDV genome using pre-validated primer–probe sets supplied with the assay kit, ensuring standardized amplification performance. The assay also includes internal controls and quantification standards for viral load estimation. All procedures were conducted according to the manufacturer’s instructions.

The qPCR reaction was prepared in a final volume of 20 µL per reaction, comprising 10 µL of extracted nucleic acid template, 5 µL of reconstituted oasig™ master mix (PrimerDesign Limited, Manchester, UK), and 5 µL of PBFDV-specific primer–probe mix. An internal control was included in each reaction to monitor nucleic acid extraction efficiency and identify potential PCR inhibition. Positive and negative controls supplied with the kit were included in each run to validate assay performance.

Amplification and fluorescence detection were carried out in the Genesig q16 Real-Time PCR instrument (PrimerDesign Limited, Manchester, UK) under the following thermal cycling conditions: an initial enzyme activation step at 95 °C for 10 min, followed by 40 amplification cycles consisting of denaturation at 95 °C for 10–30 s and annealing/extension at 60 °C for 60 s. Fluorescence signals were measured at the end of each annealing/extension step, and amplification data were collected and analyzed automatically using the Genesig q16 software.

The cycle quantification (Cq) value, defined as the cycle at which fluorescence exceeds the threshold level, was used to determine the presence of the viral DNA. The Genesig q16 Real-Time PCR platform (PrimerDesign Ltd., Manchester, UK) has been reported to achieve high analytical sensitivity, with manufacturer specifications indicating sensitivity down to approximately 1 copy of target nucleic acid under optimized assay conditions. In this present study, samples with Cq values ≤ 39 were interpreted as PBFDV DNA detected, whereas samples with Cq values > 39 were interpreted as PBFDV DNA not detected. Viral load values were quantified by comparison with a standard curve generated from known concentrations of PBFDV DNA provided with the assay kit and were expressed as estimated viral genome copy numbers per qPCR reaction. All samples were analyzed in duplicate, and reactions showing a variation greater than one Cq value between replicates were repeated to ensure data reliability. Because swab sampling targeted accumulated material within air conditioning components rather than a defined surface area, the results were interpreted as relative indicators of environmental contamination rather than absolute surface concentrations.

### 2.4. Statistical Analysis

All statistical analyses were performed using the Statistical Package for the Social Sciences (SPSS) software (version 26.0; IBM Corp., Armonk, NY, USA). Descriptive statistics were used to summarize the distribution of PBFDV viral loads across different sampling locations and room categories. Viral load data were expressed as genome copy numbers and presented as median and range due to their non-normal distribution. Viral load was categorized into three levels (low, moderate, and high) based on predefined thresholds (<100, 100–10,000, and >10,000 copies, respectively). The association between contamination level and room category was evaluated using a two-sided Fisher’s exact test due to the small sample size. Differences in viral load among multiple room categories were assessed using the Kruskal–Wallis test.

For comparative analysis based on animal type, rooms were further classified into those designated for exotic pet patients and those used for small animal practice (dogs and cats). Differences in viral load between these two groups were analyzed using the Mann–Whitney U test. In addition, the association between viral load categories (low, moderate, and high) and room type was evaluated using Fisher’s exact test. A *p*-value of less than 0.05 was considered statistically significant.

## 3. Results

### 3.1. Descriptive Statistics of PBFDV Viral Load Distribution

PBFDV DNA was detected in all sampled air conditioning systems (17/17, 100%), indicating widespread environmental contamination throughout the veterinary hospital as shown in Table 1. Viral loads varied markedly across sampling locations, ranging from 1 to 26,172 genome copies per reaction. Overall, the median viral load across all rooms was approximately 223 genome copies per reaction. The highest viral loads were identified in examination room 1 (26,172 copies) and surgical room 1 (25,730 copies), followed by examination room 3 (9996 copies). In contrast, minimal viral loads were observed in several locations, including the nebulization room (1 copy), surgical room 2 (4 copies), and examination room 6 (6 copies). These findings demonstrate a highly heterogeneous distribution of PBFDV contamination within the facility.

### 3.2. Comparison of Viral Load Categories Across Functional Areas

**Figure 2:**
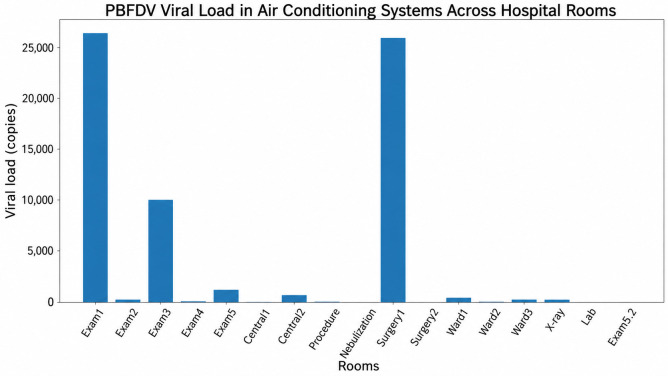
Distribution of Psittacine beak and feather disease virus (PBFDV) viral load detected in air conditioning systems across different rooms of an exotic veterinary hospital. Viral loads are expressed as genome copy numbers obtained by quantitative real-time PCR (qPCR).

When viral loads were categorized into low (<100 copies), moderate (100–10,000 copies), and high (>10,000 copies) levels, variation in contamination intensity was observed across different functional areas (Table 1). Of the 17 rooms evaluated, 8 (47.1%) were classified as low contamination, 7 (41.2%) as moderate contamination, and 2 (11.8%) as high contamination. High contamination levels were exclusively observed in clinical and surgical areas, specifically examination room 1 and surgical room 1. Moderate contamination levels were distributed across clinical, hospitalization, and support areas, whereas low contamination levels were more frequently identified in support and non-clinical rooms (Figure 2).

However, statistical analysis using Fisher’s exact test revealed no significant association between contamination level and functional room category (*p* = 0.68). Similarly, comparison of viral load as a continuous variable among functional areas using the Kruskal–Wallis test showed no statistically significant difference (*p* = 0.24), although a tendency toward higher viral loads in clinical and surgical areas was observed.

### 3.3. Comparative Analysis Based on Animal Type (Exotic vs. Small Animal Rooms)

For analysis based on animal type, rooms were classified into those designated for exotic pet patients and those used for small animal practice (dogs and cats). Viral loads in rooms designated for exotic pets were consistently higher than those observed in small animal rooms. The median viral load in exotic pet rooms was 9996 copies (range: 426–26,172), whereas the median viral load in small animal rooms was 61.5 copies (range: 4–223). Statistical comparison using the Mann–Whitney U test demonstrated that viral loads were significantly higher in exotic pet rooms than in small animal rooms (*p* = 0.02).

In addition, when viral load was categorized into low, moderate, and high levels, all exotic pet rooms were classified within the moderate-to-high contamination range, whereas the majority of small animal rooms were categorized as low contamination. This difference in distribution was statistically significant (Fisher’s exact test, *p* = 0.03). These findings indicate that rooms designated for exotic pet patients represent a major source of environmental PBFDV contamination within the hospital, supporting the hypothesis that host type influences viral load distribution in clinical environments.

## 4. Discussion

The present study demonstrates widespread environmental contamination with PBFDV within air conditioning systems of a veterinary hospital, with all sampled locations testing positive for viral DNA. This finding highlights the important role of potential environmental sources in the maintenance and dissemination of PBFDV in clinical settings. The detection of viral DNA across multiple room types, including non-clinical areas, reflects the notable environmental persistence of circoviruses [12,13,14]. This stability may allow viral particles to persist in organic materials such as feather dust and fecal debris, potentially contributing to indirect transmission within enclosed environments [4].

Evidence from field studies further supports the importance of environmental persistence in PBFDV transmission dynamics. Nest cavities used by wild psittacine birds have been identified as significant environmental reservoirs, where viral DNA can remain detectable for up to 3.7 months after birds have vacated the site, and contamination rates may reach approximately 80% in nests associated with actively shedding individuals [15]. These findings provide important insights into the environmental factors contributing to viral spread, including repeated use of confined spaces, accumulation of contaminated organic material, and the intensity of host occupancy. In veterinary hospital settings, similar conditions may promote environmental contamination, while additional factors such as air circulation systems may further facilitate the redistribution of viral particles across different areas [16,17]. Together, these observations underscore the complex interplay between viral persistence, host activity, and environmental conditions in shaping PBFDV transmission and highlight the need for effective environmental control and biosecurity measures. A key finding of this study is the significantly higher viral load observed in rooms designated for exotic pet patients compared to those used for small animal practice. This observation is consistent with the known host specificity of PBFDV, which primarily infects psittacine birds [18,19]. The elevated viral loads in exotic animal areas likely reflect active viral shedding from infected or carrier birds, particularly through feather dust and dander, which are recognized as major sources of viral dissemination [15].

Notably, rooms with the highest viral loads may function as potential environmental sources of PBFDV within the hospital setting. In such areas, continuous shedding and accumulation of virus-laden particulates can increase the likelihood of environmental persistence and secondary dissemination. Given that air conditioning systems recirculate indoor air, these contaminated particles may potentially be redistributed to adjacent areas within the facility through routine air circulation. However, the present study did not directly assess airflow dynamics, viral viability, or airborne transmission pathways. This mechanism may explain the detection of PBFDV DNA in rooms dedicated to dogs and cats, despite the absence of susceptible host species, and highlights a possible association between ventilation systems and the environmental distribution of viral DNA within veterinary hospitals. This observation is supported by previous studies demonstrating that viral pathogens can be disseminated through air conditioning and ventilation systems, contributing to airborne transmission and environmental spread within indoor environments [20,21]. The role of aerosolized particles and ventilation systems in pathogen transmission has gained increasing attention in both veterinary and human healthcare settings. Air conditioning systems, particularly those that recirculate indoor air, may facilitate the distribution of infectious particles across different areas of a facility [22]. Previous research indicated that mechanical ventilation, particularly systems that recirculate air, can act as a vector by transporting infectious bioaerosols across different zones of a facility [23,24]. In the context of PBFDV, which can be transmitted via inhalation of contaminated feather dust and aerosols, such systems may be associated with the environmental dispersal of aerosol-associated viral particles, contributing to the spread of viral particles beyond the immediate vicinity of infected birds [14]. The detection of PBFDV in air conditioning units in the present study supports the possibility that airborne environmental contamination may contribute to the distribution of viral particles within enclosed clinical environments. However, direct airborne transmission was not evaluated in the present study.

From a One Health perspective, these findings have broader implications for infection control and environmental health. Veterinary hospitals represent interfaces where animal, human, and environmental health intersect, and the presence of persistent viral contaminants in shared air systems raises concerns regarding cross-species exposure and occupational health risks [25]. Importantly, such contamination is not limited to PBFDV alone but may also involve other viral pathogens capable of airborne transmission or environmental persistence, including those that can adhere to feather dust and organic debris and subsequently become aerosolized [26]. In avian species, feather-associated particles have been recognized as effective carriers of viral agents, facilitating their dissemination within enclosed environments [27]. These characteristics underscore the broader relevance of environmental contamination in veterinary settings, where airborne or particulate-associated pathogens may circulate between animal hosts, the environment, and potentially humans, reinforcing the importance of integrated One Health-based infection control strategies. In addition to environmental contamination, such conditions may facilitate nosocomial transmission among animal patients, particularly when susceptible individuals are exposed to contaminated air or surfaces within the clinical setting [28]. Although PBFDV is not considered zoonotic, the mechanisms of environmental persistence and aerosol dissemination observed in this study are relevant to other pathogens of public health importance, including those capable of interspecies transmission. Therefore, strengthening environmental surveillance and biosecurity measures in veterinary facilities is essential not only for animal health but also for protecting staff health and animal owners in cases involving zoonotic agents. The absence of statistically significant differences in viral load among functional room categories (e.g., clinical vs. non-clinical areas) suggests that environmental contamination may not be confined to high-risk zones alone. This finding aligns with previous studies demonstrating that pathogens can disseminate throughout healthcare environments via indirect routes, including air flow, fomites, and human movement [29]. Consequently, infection control strategies should not be limited to areas of direct animal contact but should encompass the entire facility, including ventilation and air handling systems.

It is important to note that qPCR-based detection identifies viral nucleic acids but does not distinguish between infectious and non-infectious viral particles. Therefore, the presence of PBFDV DNA in air conditioning systems should be interpreted as evidence of environmental contamination rather than confirmed infectivity or transmission risk. In addition, this study was based on single-time-point sampling per location, which limits the ability to assess temporal variation in environmental contamination. Despite these limitations, the findings provide valuable baseline data on environmental contamination within veterinary hospital settings. Future studies incorporating viral viability assays and longitudinal sampling are required to better understand the epidemiological significance of these observations. In addition, the relatively small sample size may have limited the statistical power of the analyses and increased the possibility of false-negative findings, particularly for non-parametric statistical tests. Consequently, the results should be interpreted as preliminary observations derived from a single-center environmental surveillance study.

Future research should focus on longitudinal monitoring of PBFDV contamination within veterinary hospital environments to better understand temporal patterns of viral persistence and the effectiveness of intervention strategies over time. In addition, assessing viral viability, rather than relying solely on nucleic acid detection, would provide more meaningful insights into the actual infectious risk associated with environmental contamination. Although molecular methods such as PCR offer high sensitivity for detecting viral DNA or RNA, they are unable to differentiate between infectious viral particles and non-viable genetic remnants [30]. Expanding this approach to multiple veterinary facilities would further strengthen the external validity of the findings and allow for comparative evaluation of environmental transmission dynamics across different clinical settings [31]. Importantly, the findings of the present study provide a practical framework for the development of targeted infection prevention and control strategies. The application of qPCR-based environmental surveillance, particularly through swab sampling of air conditioning systems, offers a sensitive and reliable method for detecting and quantifying PBFDV contamination within clinical environments. Such data can be used to identify high-risk areas and guide evidence-based interventions aimed at reducing viral load. Recommended measures include regular and systematic cleaning of air conditioning units and surrounding surfaces using appropriate disinfectants, routine removal of organic debris such as feather dust, and the implementation of enhanced air filtration systems to reduce airborne particle dissemination [32,33].

Furthermore, infection control strategies should adopt a holistic approach by integrating environmental management with clinical practices, including the segregation of high-risk species, routine screening of avian patients using molecular diagnostics, and adherence to strict biosecurity protocols. Although such measures may involve increased operational costs, their implementation is essential for minimizing the risk of environmental contamination and nosocomial transmission. Cooperatively, this study demonstrates that environmental monitoring using qPCR can serve not only as a diagnostic tool but also as a practical component of infection control programs, ultimately contributing to improved avian health management and safer veterinary healthcare environments.

## 5. Conclusions

This study demonstrates the widespread presence of Psittacine beak and feather disease virus (PBFDV) DNA within air conditioning systems of a veterinary hospital, highlighting the presence of environmental contamination and suggesting a potential role in the environmental distribution of PBFDV, although infectivity and transmission were not assessed in this study. The markedly higher viral loads observed in rooms designated for exotic pet patients suggest that these areas may contribute to potential environmental sources, where continuous viral shedding leads to the accumulation and persistence of virus-associated particulates. The detection of PBFDV DNA in rooms without susceptible host species further indicates that indirect transmission pathways, particularly those involving aerosolized particles and air recirculation systems, may contribute to the distribution of viral contaminants within the hospital environment.

These findings emphasize the value of incorporating environmental surveillance into routine infection control practices in veterinary hospitals. The use of qPCR-based monitoring provides a sensitive and practical approach for identifying areas of contamination and supporting targeted interventions. Although PBFDV is not considered a zoonotic pathogen, the mechanisms of environmental persistence and airborne dissemination observed in this study are relevant to other infectious agents of veterinary and public health significance. Strengthening biosecurity practices, including regular cleaning and disinfection, appropriate maintenance of air handling systems, and risk-based spatial management, is therefore essential to reduce environmental contamination, limit nosocomial transmission among animal patients, and protect the health of veterinary staff.

## Figures and Tables

**Table 1:** PBFDV contamination levels and qPCR (Cq) results in air conditioning systems across functional areas of a veterinary hospital.

Room	Functional Area	Risk Category	Viral Load (Copies)	Contamination Level	Cq (PBFDV)	Cq (Internal Control)	PBFDV Detection
Examination room 1	Clinical	High-risk	26,172	High	21.66	24.54	Detected
Examination room 2	Clinical	High-risk	143	Moderate	28.65	24.78	Detected
Examination room 3	Clinical	High-risk	9996	Moderate	22.52	24.57	Detected
Examination room 4	Clinical	High-risk	55	Low	30.02	23.31	Detected
Examination room 5	Clinical	High-risk	1135	Moderate	26.19	24.54	Detected
Examination room 6	Clinical	High-risk	6	Low	33.12	25.80	Detected
Central area 1	Support	Low-risk	22	Low	31.37	26.26	Detected
Central area 2	Support	Low-risk	661	Moderate	26.19	24.54	Detected
Procedure room	Clinical	High-risk	41	Low	30.44	24.79	Detected
Nebulization room	Clinical	High-risk	1	Low	38.21	25.29	Detected
Surgical room 1	Surgical	High-risk	25,730	High	21.68	26.04	Detected
Surgical room 2	Surgical	High-risk	4	Low	33.97	25.20	Detected
Ward 1	Hospitalization	Moderate-risk	426	Moderate	27.37	24.32	Detected
Ward 2	Hospitalization	Moderate-risk	68	Low	30.01	24.64	Detected
Ward 3	Hospitalization	Moderate-risk	223	Moderate	28.31	24.59	Detected
X-ray room	Support	Low-risk	197	Moderate	28.48	25.23	Detected
Laboratory	Support	Low-risk	9	Low	33.84	25.32	Detected

## Data Availability

The original contributions presented in this study are included in the article. Further inquiries can be directed to the corresponding author.
